# Submucosal small-cell neuroendocrine carcinoma of the larynx detected using ^18^F-fluorodeoxyglucose positron emission tomography/computed tomography: A case report and review of the literature

**DOI:** 10.3892/ol.2014.2246

**Published:** 2014-06-12

**Authors:** HONG-FANG YING, YANG-YANG BAO, SHUI-HONG ZHOU, LIANG CHAI, KUI ZHAO, TING-TING WU

**Affiliations:** 1 Department of Otolaryngology, The First Affiliated Hospital, College of Medicine, Zhejiang University, Hangzhou, Zhejiang 310003, P.R. China; 2 The PET Center, The First Affiliated Hospital, College of Medicine, Zhejiang University, Hangzhou, Zhejiang 310003, P.R. China

**Keywords:** small-cell neuroendocrine carcinoma, unknown primary cancers, larynx, ^18^F-fluorodeoxyglucose positron emission tomography/computed tomography

## Abstract

A 67-year-old male presented with a metastatic carcinoma in the right side of the neck from an unknown primary site. ^18^F-fluorodeoxyglucose (FDG) positron emission tomography/computed tomography showed increased ^18^F-FDG uptake in the right larynx and right neck lymph nodes. A smooth lesion was identified in the submucosa of the right supraglottic region via a suspension laryngoscopy under general anaesthesia. A biopsy was performed and a frozen section revealed a small-cell (SC) carcinoma. A total laryngectomy and bilateral neck dissection were performed simultaneously, and the pathological results demonstrated a SC neuroendocrine carcinoma. The patient received chemo-radiotherapy postoperatively, however, succumbed due to distant metastasis one year following surgery.

## Introduction

Neuroendocrine carcinomas (NECs) account for <5% of unknown primary cancers (CUPs) worldwide (1). A primary site in the larynx is rare; Hess *et al* (2) reported 43 patients with NEC in 1,000 CUP patients and there was no case of a carcinoma in the larynx. To date, there are ~520 cases of NEC with CUP in the English-language literature (3–6) and to the best of our knowledge, there is only one previously reported case of a CUP of NEC from the larynx (6).

Detection of the carcinoma origin in CUP patients is a challenge. Although the primary sites may be identified by conventional modalities, including complete physical examination, panendoscopy and conventional imaging (including computed tomography [CT], magnetic resonance imaging [MRI] and even random biopsies (including, tonsillectomies) commonly, the origin remains undetected using the conventional diagnostic procedures (7).

Accurate identification of the unknown primary site is important, as it enables the therapy to be focused towards the known site of origin, thus, decreasing treatment-associated morbidity and improving therapeutic efficacy (9,10). ^18^F-fluorodeoxyglucose (FDG) positron emission tomography (PET)/CT has provided novel insights in the diagnosis of CUP (8,9). However, there have been few studies regarding the effectiveness of ^18^F-FDG PET/CT in detecting the CUP in NEC (4–6,10).

In the present study, we report a case of cervical metastatic small-cell (SC) NEC of CUP and detected the origin in the larynx using ^18^F-FDG PET/CT. The patient’s family provided written informed consent.

## Case report

### Patient

A 67-year-old male presented to the Department of Otolaryngology, The First Affiliated Hospital, College of Medicine, Zhejiang University (Hangzhou, China), on August 30, 2010 with a one-month history of a progressively enlarging mass in the right side of the neck. The patient’s medical history was unremarkable, although he was a heavy smoker. The ear, nose and throat examination was normal, which included a nasoendoscopy and laryngoscopy. A CT scan of the neck revealed no additional abnormal finding other than the lymphadenopathy of the right neck. MRI of the nasopharynx identified abnormal lesions in the nasopharynx and a biopsy of the lymphadenopathy in the right upper neck indicated a poorly differentiated metastatic carcinoma; a nasopharyngeal biopsy did not detect carcinoma cells. Furthermore, a pulmonary CT and gastroscopy did not reveal any abnormal finding in the lung or stomach. Subsequently, ^18^F-FDG PET/CT was performed.

### PET/CT

Whole-body imaging was conducted using a combined PET/CT scanner (Biograph Sensation 16. LSO 39-ring; Siemens Medical, Erlangen, Germany). Following ≥4–6 h of fasting, the patient received an intravenous injection of ^18^F-FDG at 5.5–7.4 MBq (0.15–0.20 mCi)/kg body weight. Patient blood glucose levels were assessed prior to the ^18^F-FDG injection. Data acquisition for the diagnostic CT commenced 60–90 min prior to ^18^F-FDG administration. The data acquisition procedure was as follows: i) A 16-section multi-detection row CT scan was performed from head to mid-thigh at 120 kV, 50 mAs. ii) Using a tube rotation time of 0.5 sec, a 2–5-mm thick section was matched to the PET section thickness. iii) Finally, a three-dimensional PET was conducted with the patient in the same supine position. The PET scan incorporated the subcranial region to the mid-thigh; however, the brain scan required an additional bed position. The acquisition time was 2 min per bed position. The total imaging time of the PET/CT study was ~20 min. Attenuation correction was based on the CT scan. The PET images were reconstructed iteratively using the ordered subset Syngo Speaking software (Wizard Workstation; Siemens Medical). PET, CT and fused PET/CT images were generated and reviewed on a computer; the co-registered images were displayed on a workstation. The PET/CT scans were interpreted independently by two experienced members of our PET centre who were unaware of the histology of the metastatic sites. Any differences in their interpretations were settled by consensus with a final unanimous opinion. The standardised uptake value (SUV) was collected from the predominant lesion and calculated based on the attenuation-corrected images, amount of injected ^18^F-FDG and patient body weight: SUV_max_ = [decay corrected activity (kBq)/tissue volume (ml)]/[injected ^18^F-FDG activity (kBq)/body weight (g)]. When multiple lymph nodes were found, only the lymph node with the greatest SUV_max_ was used.

**Figure 1:**
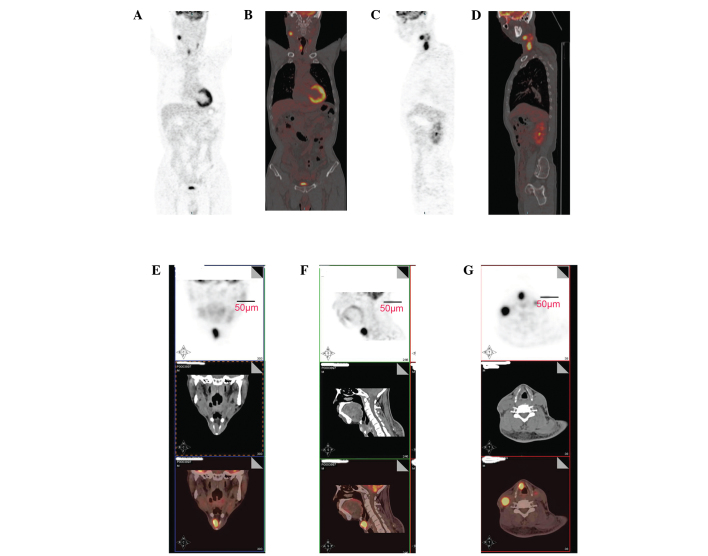
^18^F-fluorodeoxyglucose positron emission tomography/computed tomography (^18^F-FDG PET/CT) was performed. Whole body images of ^18^F-FDG PET/CT and fused PET/CT images in the (A,B) coronal and (C, D) sagittal plane, in addition to local images of ^18^F-FDG PET/CT, CT and fused PET/CT images in the (E) coronal, (F) sagittal and (G) axial plane demonstrated increased ^18^F-FDG uptake in the right larynx (SUV_max_ = 8.37), right neck lymph nodes (SUV_max_ = 11.5) and no distant metastasis.

PET/CT showed increased ^18^F-FDG uptake in the right larynx (SUV_max_ = 8.37) and right neck lymph nodes (SUV_max_ = 11.5; Fig. 1). These findings indicated the possibility of a primary laryngeal carcinoma.

### Surgery

A smooth lesion was observed in the submucosa of the right laryngeal ventricle and the right false vocal cord using suspension laryngoscopy under general anaesthesia. A deep biopsy was performed and a frozen section revealed a laryngeal SC carcinoma. The diagnosis was supraglottic laryngeal carcinoma (T_2_N_2C_M_0_), clinical stage IV according to the tumor, lymph node, metastases (TNM) staging system (11). A total laryngectomy and bilateral neck dissection were performed simultaneously. During surgery, the largest lymph node observed in the left neck was ~3.0×1.8×1.6 cm, and multiple small lymph nodes were identified around the left jugular vein and carotid artery. These lymph nodes did not adhere to the blood vessels above. A selective neck dissection of the left II, III, IV and V regions, preserving the left jugular vein, sternocleidomastoid muscle and accessory nerve, was performed. In addition, multiple lymph nodes were observed in the right neck around the jugular vein and carotid artery. The largest was ~5.2×3.8×3.5 cm, somewhat adherent to the right carotid artery and surrounded the right jugular vein tightly. A selective neck dissection of the right II, III, IV and V regions and dissection of the right jugular vein and sternocleidomastoid muscle, with preservation of the accessory nerve, was performed. A prelaryngeal lymph node of ~5×4×3.4 mm was dissected simultaneously. The primary lesion in the larynx (size, ~2.5×2.3×1.8 cm) was a smooth submucosal lesion, which was located predominantly in the right supraglottic area and involved the preepiglottic space, right false vocal cord and laryngeal ventricle. The postoperative period was uneventful.

### Pathological and immunohistochemical findings

**Figure 2:**
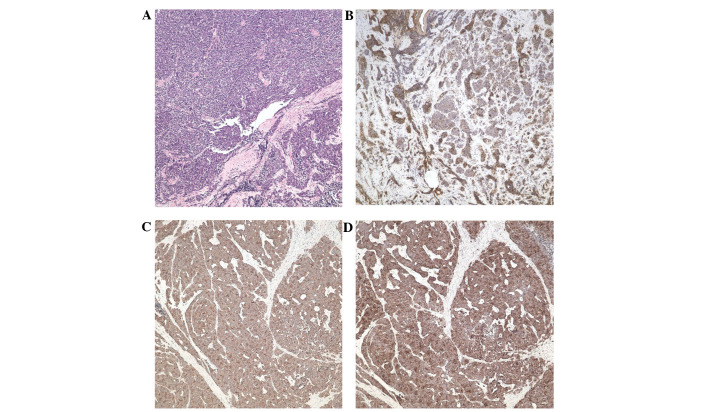
(A) Microscopically, the lesions were composed of sheets of small atypical cells with little cytoplasm and large, round nuclei (stain, hematoxylin and eosin; magnification, ×100). Immunohistochemical EnVision™ staining revealed that the neoplastic cells were positive for (B) cytokeratins, (C) synaptophysin and (D) chromogranin A (magnification, ×100).

Microscopically, the lesions were composed of sheets of small atypical cells with sparse cytoplasm and large, round nuclei (Fig. 2A). The immunohistochemical results revealed that the neoplastic cells were positive for cytokeratins (Fig. 2B), synaptophysin (Fig. 2C) and chromogranin A (Fig. 2D), and negative for neuron-specific enolase, vimentin, desmin and S-100. These resulted indicated SCNEC in the larynx. Five lymph nodes in the right neck, one lymph node in the left neck and one prelaryngeal lymph node were positive for metastatic SCNEC. The pathological TNM stage was pT_2_N_2_M_0_ and the resection margins were negative.

### Follow-up

The patient received concurrent chemoradiotherapy postoperatively, however, succumbed due to distant metastasis one year following surgery.

## Discussion

NEC of CUP is rare, and its diagnosis and therapy are complex. Stoyianni *et al* (12) systematically reviewed all English-language publications studying neuroendocrine CUP patients, and identified only 500 cases. Since that study, an additional 20 NEC patients with CUP have been reported (3–6). To the best of our knowledge, there is only one reported case of CUP of NEC from the larynx (6). NEC of CUP has a particularly poor outcome. If the primary site is detected and the metastasis is confined to a single site, surgery or radiation therapy may be conducted and can occasionally be curative (1).

Laryngeal NEC is rare and constitutes <1% of all tumours originating from the larynx (13). Laryngeal SCNEC, located in the submucosa of the larynx (14), and approximately half of all laryngeal SCNEC occur as cervical lymphadenopathy; however, the primary lesion may be difficult to detect as CUP (6). PET/CT has been used widely in the detection of CUP; however, there have been few reports concerning the detection of CUP of NEC using PET/CT (3–6). Naswa *et al* (5) detected the primary tumour in 12/20 (60%) patients with CUP of NEC using ^68^Ga-DOTA-NOC PET/CT. In addition, Prasad *et al* (10) demonstrated that ^68^Ga-DOTA-NOC PET/CT localised the primary tumour in 59% of patients. The management of 15 and 10% of patients in the two reports, respectively, was modified upon identifying the primary tumour. The authors proposed that ^68^Ga-DOTA-NOC PET/CT had particularly high sensitivity and specificity in the detection of NECs. Certain studies have shown that ^18^F-FDG PET/CT is able to detect unknown primary tumours and additional metastatic sites in patients with CUP (9,15). However, Adams *et al* (16) also identified false-negative results in the detection of 15 NECs due to a low rate of tumour glucose metabolism. Thus, whether there is high uptake of ^18^F-FDG in the NECs remains controversial. In the present case, we detected high ^18^F-FDG uptake in the larynx and cervical lymph nodes. High ^18^F-FDG uptake in cancer lesions (including NEC) is associated with the overexpression of glucose transporter-1 (GLUT-1) (17). In the present case, GLUT-1 protein expression was identified as positive by immunohistochemistry. In our previous case report, high ^18^F-FDG uptake in the cervical lymph nodes and lungs was observed in a patient with tonsillar metastasis from a lung SCNEC (18). Furthermore, Miki *et al* (6) reported high ^18^F-FDG uptake in a case of a metastatic cervical SCNEC from the larynx by ^18^F-FDG PET/CT. In addition, high ^18^F-FDG uptake has been detected in metastatic NECS from other sites (19–22). Song *et al* (17) found that ^18^F-FDG uptake was correlated with GLUT-1 expression in 32 lung NECs. Thus, whether there is low or high glucose metabolism in NECs requires further investigation according to the NEC type.

Laryngeal NECs usually arise submucosally and may be too small to be detected by PET (6). Therefore, the performance of deeper submucosal biopsies is required once NECs have been detected via radiological imaging. SCNECs are particularly aggressive tumours. Approximately 50% of all SCNEC patients present with cervical lymph node metastases and >90% of patients with this type of tumour develop metastatic disease (23). Laryngeal SCNEC is located predominantly in the supraglottis and its metastasis to neck lymph nodes always occurs earlier than that in the glottic area and unilaterally. In the present case, bilateral metastatic cervical lymph nodes (including the prelaryngeal lymph node) were present, which were demonstrated by the pathological examination.

There is no specific treatment for NEC of the larynx. Patients may benefit from surgery; however, radiotherapy and chemotherapy remain the treatments of choice (14). However, surgical management of laryngeal SCNEC is not as effective. Radical surgical procedures (including total laryngectomy and radical neck dissection) have failed in the majority of reported cases (24). A total laryngectomy was performed in the present case and the patient received concurrent chemoradiotherapy postoperatively. However, the outcome was unfavourable as the patient succumbed due to distant metastasis one year after surgery, which is similar to a previous report (6). As a result of the current case, it is proposed that frozen sections should not be obtained during surgery and that the pathological type of the laryngeal tumour requires accurate identification preoperatively, using routine pathological examinations, including immunohistochemistry. Once the diagnosis of laryngeal SCNEC is established, concurrent chemoradiotherapy is considered to be the optimum treatment regimen (24). As early as 1986, Baugh *et al* (25) found that the combination of radiotherapy and chemotherapy resulted in a significantly longer survival time compared with any other treatment regimen. In the same year, Ferlito *et al* (26) reported a case of laryngeal SCNEC, where the patient was free of disease for more than five years following treatment with chemotherapy and radiotherapy. Recently, definitive chemoradiotherapy was associated with better outcomes in extrapulmonary SC cancers (27). Thus, it is proposed that the therapeutic methods for laryngeal SCNEC should not follow those of squamous cell carcinoma as others have recommended (14) and, instead, laryngeal SCNEC should be considered as a systemic disease, as SC lung cancer is (24).

In conclusion, the present study is the second case of a CUP of NEC from the larynx. ^18^F-FDG PET/CT is considered to be an effective work-up for the detection of CUP of NEC. As a result of the present case, it is proposed that frozen sections should not be obtained during surgery and that the pathological type of the laryngeal tumour should be accurately established preoperatively via routine pathological examinations, including immunohistochemistry. Once the diagnosis of laryngeal SCNEC is established, concurrent chemoradiotherapy is considered to be the optimum treatment regimen.
